# Association of *CKIP-1* P21A polymorphism with risk of chronic heart failure in a Chinese population

**DOI:** 10.18632/oncotarget.16614

**Published:** 2017-03-28

**Authors:** Mu-Peng Li, Yan-Jiao Zhang, Xiao-Lei Hu, Ji-Peng Zhou, Yong-Long Yang, Li-Ming Peng, Hong Qi, Tian-Lun Yang, Xiao-Ping Chen

**Affiliations:** ^1^ Department of Clinical Pharmacology, Xiangya Hospital, Central South University, Changsha 410008, Hunan, P. R. China; ^2^ Institute of Clinical Pharmacology, Central South University, Hunan Key Laboratory of Pharmacogenetics, Changsha 410078, Hunan, P. R. China; ^3^ Haikou People's Hospital and Affiliated Haikou Hospital of Xiangya Medical School, Central South University, Haikou 570311, Hainan, P. R. China; ^4^ Department of Cardiovascular Medicine, Xiangya Hospital, Central South University, Changsha 410008, Hunan, P. R. China

**Keywords:** CKIP-1, polymorphism, cardiac hypertrophy, chronic heart failure, prognosis

## Abstract

Pathological cardiac hypertrophy is an independent risk factor for chronic heart failure. Casein kinase-2 interacting protein-1 (CKIP-1) can inhibit pathological cardiac hypertrophy. Therefore, we investigated whether *CKIP-1* nonsynonymous polymorphism rs2306235 (Pro21Ala) contributes to risk and prognosis of chronic heart failure in a Chinese population.A total of 923 adult patients with chronic heart failure and 1020 age- and gender-matched healthy controls were recruited. *CKIP-1* rs2306235 polymorphism was genotyped using PCR-restriction fragment length polymorphism. Additional follow-up data for 140 chronic heart failure patients was evaluated. The rs2306235 G allele was associated with an increased risk of chronic heart failure (OR = 1.38, 95% CI = 1.09-1.75, *p* = 0.007), especially in patients with hypertension (OR = 1.45, 95% CI = 1.09-1.75, *p* = 0.006) and coronary heart disease (OR = 1.41, 95% CI = 1.09-1.83, *p* = 0.010) after adjustment for multiple cardiovascular risk factors. However, rs2306235 polymorphism was not associated with cardiovascular mortality in chronic heart failure (*p* = 0.875). *CKIP-1* rs2306235 polymorphism may be a risk factor for chronic heart failure in a Chinese Han population.

## INTRODUCTION

Chronic heart failure (CHF) is a complex clinical syndrome with a multifactorial etiology including genetic factors [1–6]. Nevertheless, the genetic background for sporadic CHF is poorly characterized yet, and identification of novel CHF susceptibility loci is helpful for personalized diagnosis and treatment of the disease.

The pleckstrin homology domain-containing protein casein kinase-2 interacting protein-1 (CKIP-1) was originally identified as a casein kinase-2 α-subunit (CK2α) interacting protein [7]. CKIP-1 protein contains a pleckstrin homology (PH) domain and a leucine zipper (LZ) motif, which could mediate multiple interactions between CKIP-1 and numerous cellular proteins [8]. CKIP-1 plays scaffold roles in various signaling pathways [8], controlling cell growth [9–13], apoptosis [14, 15], differentiation [16], migration [17], morphology and cytoskeleton [18–20], and bone formation [21]. Moreover, CKIP-1 can inhibit pathological cardiac hypertrophy by promoting dephosphorylation of histone deacetylase 4 (HDAC4) through recruiting serine/threonine protein phosphatase 2A (PP2A) [22]. In mice model, cardiac-specific deficiency and overexpression of *CKIP-1* exhibit hypersensitivity and resistance to pathological cardiac hypertrophy induced by pressure overload, respectively [22]. Also, CKIP-1 protein level was sharply reduced in the hypertrophied failing human hearts [22].

Pathological cardiac hypertrophy is an independent risk factor for CHF. Considering the crucial role of CKIP-1 in inhibiting pathological cardiac hypertrophy, *CKIP-1* may represent a potential candidate gene for CHF. However, there is no report about association between *CKIP-1* polymorphisms and risk of CHF yet. By using the 1000 genome data, we observed rs2306235 polymorphism (Pro21Ala) in *CKIP-1*, and the 21st amino acid locates in the PH domain of CKIP-1. The PH domain is essential for localization of CKIP-1 in the plasma membrane (PM) and recruitment of multiple proteins to the PM [23]. To make clear the clinical relevance of *CKIP-1* rs2306235 polymorphism, we herein performed a case-control study to investigate the association between *CKIP-1* rs2306235 polymorphism and risk of CHF in a Chinese population.

## RESULTS

### Baseline characteristics of study participants

Baseline characteristics of the study participants were shown in Table 1. CHF and control participants were well matched in gender and age. The coefficients of variation, skewness, and kurtosis of healthy controls’ age were 13.6%, -0.6, and 0.6, respectively. The coefficients of variation, skewness, and kurtosis of patients’ age were 17.9%, -0.9, and 0.8, respectively. On the basis of selection criteria, controls had no hypertension, coronary heart disease, and diabetes. More participants in CHF group had dyslipidemia, smoking habit, higher levels of systolic and diastolic blood pressure, total cholesterol, triglyceride, low-density lipoprotein cholesterol, but lower level of high-density lipoprotein cholesterol. Etiologies of CHF consisted of 74.9% ischemic and 25.1% nonischemic heart failure.

**Table 1:** Baseline characteristics of the study population

Characteristics	CHF (n = 923)	Control (n = 1020)	*P*
Male, n (%)	564 (61.1)	600 (58.8)	0.305
Age (years)	61 ± 11	60 ± 8	0.122
SBP (mmHg)	133.4 ± 25.6	113.6 ± 9.5	<0.001
DBP (mmHg)	79.4 ± 14.4	72.9 ± 6.7	<0.001
TC (mmol/L)	4.6 ± 1.0	3.9 ± 1.4	<0.001
TG (mmol/L)	1.8 ± 1.8	1.5 ± 1.3	0.001
HDL-C (mmol/L)	1.3 ± 1.0	1.8 ± 0.7	<0.001
LDL-C (mmol/L)	2.4 ± 0.9	2.1 ± 0.6	<0.001
Cigarette smoker, n (%)	313 (33.9)	255 (25.0)	<0.001
Dyslipidemia, n (%)	267 (28.9)	196 (19.2)	<0.001
Hypertension, n (%)	597 (64.7)	0 (0)	<0.001
Coronary heart disease, n (%)	691 (74.9)	0 (0)	<0.001
Diabetes mellitus (%)	233 (25.2)	0 (0)	<0.001
Family history of heart failure, n (%)	3 (0.3)	-	
Etiology of heart failure			
Ischemic, n (%)	691 (74.9)	-	
Nonischemic, n (%)	232 (25.1)	-	
Medications			
Beta-blockers, n (%)	720 (78.0)	-	
ACE inhibitors, n (%)	474 (51.3)	-	
Angiotensin receptor blockers, n (%)	244 (26.4)	-	
Diuretics, n (%)	212 (23.0)	-	
Digoxin, n (%)	166 (18.0)	-	
Aldosterone antagonists, n (%)	151 (16.3)	-	

SBP: systolic blood pressure, DBP: diastolic blood pressure, TC: total cholesterol, TG: triglyceride, HDL-C: high-density lipoprotein cholesterol, LDL-C: low-density lipoprotein cholesterol.

### *CKIP-1* rs2306235 polymorphism and risk of CHF

The rs2306235 genotype distribution was shown in Table 2. Rs2306235 polymorphism was in Hardy-Weinberg equilibrium in both CHF patients and control population (*p* = 0.896 and 0.728, respectively). The frequency of rs2306235 GC and GG genotype was significantly higher in CHF patients than in the controls (Table 2). Univariate analysis revealed that rs2306235 G allele (21Ala) was associated with increased risk of CHF (OR = 1.36, 95% CI = 1.07-1.71, *p* = 0.010). After adjustment for age, gender, smoking status, and dyslipidemia, the association was still significant (OR = 1.38, 95% CI = 1.09-1.75, *p* = 0.007).

**Table 2:** Association of *CKIP-1* rs2306235 polymorphism with risk of CHF

Models	Genotypes	CHF, n (%)	Control, n (%)	Unadjusted OR (95% CI)	*P*	Adjusted OR* (95% CI)	P*	
**Entire cohort**								
Additive	CC	736 (79.7)	859 (84.2)	1.00 (reference)		1.00 (reference)		
	GC	176 (19.1)	155 (15.2)	1.33 (1.05-1.68)	0.020	1.36 (1.07-1.73)	0.013	
	GG	11 (1.2)	6 (0.6)	1.46 (0.89-2.41)	0.136	1.43 (0.86-2.38)	0.163	
Dominant	CC	736 (79.7)	859 (84.2)	1.00 (reference)		1.00 (reference)		
	GC/GG	187 (20.3)	161 (15.8)	1.36 (1.07-1.71)	0.010	1.38 (1.09-1.75)	0.007	
**Hypertension**								
Additive	CC	473 (79.2)	859 (84.2)	1.00 (reference)		1.00 (reference)		
	GC	116 (19.4)	155 (15.2)	1.36 (1.04-1.77)	0.024	1.43 (1.08-1.88)	0.012	
	GG	8 (1.3)	6 (0.6)	1.56 (0.91-2.65)	0.103	1.47 (0.85-2.55)	0.166	
Dominant	CC	473 (79.2)	859 (84.2)	1.00 (reference)		1.00 (reference)		
	GC/GG	124 (20.8)	161 (15.8)	1.40 (1.08-1.81)	0.011	1.45 (1.11-1.90)	0.006
**Nonhypertension**								
Additive	CC	263 (80.7)	859 (84.2)	1.00 (reference)		1.00 (reference)		
	GC	60 (18.4)	155 (15.2)	1.26 (0.91-1.76)	0.162	1.25 (0.89-1.75)	0.199	
	GG	3 (0.9)	6 (0.6)	1.28 (0.64-2.56)	0.490	1.27 (0.62-2.61)	0.522	
Dominant	CC	263 (80.7)	859 (84.2)	1.00 (reference)		1.00 (reference)		
	GC/GG	63 (19.3)	161 (15.8)	1.28 (0.93-1.76)	0.136	1.26 (0.90-1.76)	0.174	
**CHD**								
Additive	CC	550 (79.6)	859 (84.2)	1.00 (reference)		1.00 (reference)		
	GC	133 (19.2)	155 (15.2)	1.34 (1.04-1.73)	0.025	1.39 (1.07-1.82)	0.015	
	GG	8 (1.2)	6 (0.6)	1.44 (0.85-2.46)	0.177	1.37 (0.79-2.38)	0.267	
Dominant	CC	550 (79.6)	859 (84.2)	1.00 (reference)		1.00 (reference)		
	GC/GG	141 (20.4)	161 (15.8)	1.37 (1.07-1.76)	0.014	1.41 (1.09-1.83)	0.010	
**Non-CHD**								
Additive	CC	186 (80.2)	859 (84.2)	1.00 (reference)		1.00 (reference)		
	GC	43 (18.5)	155 (15.2)	1.28 (0.88-1.86)	0.193	1.24 (0.84-1.82)	0.280	
	GG	3 (1.3)	6 (0.6)	1.52 (0.76-3.05)	0.240	1.81 (0.89-3.69)	0.103	
Dominant	CC	186 (80.2)	859 (84.2)	1.00 (reference)		1.00 (reference)		
	GC/GG	46 (19.8)	161 (15.8)	1.32 (0.92-1.90)	0.135	1.29 (0.89-1.88)	0.180	
**Diabetes**								
Additive	CC	185 (79.4)	859 (84.2)	1.00 (reference)		1.00 (reference)		
	GC	47 (20.2)	155 (15.2)	1.41 (0.98-2.02)	0.065	1.65 (1.12-2.45)	0.012	
	GG	1 (0.4)	6 (0.6)	0.88 (0.30-2.54)	0.813	0.72 (0.23-2.21)	0.561	
Dominant	CC	185 (79.4)	859 (84.2)	1.00 (reference)		1.00 (reference)		
	GC/GG	48 (20.6)	161 (15.8)	1.38 (0.97-1.98)	0.076	1.59 (1.08-2.35)	0.019	
**Nondiabetes**								
Additive	CC	551 (79.9)	859 (84.2)	1.00 (reference)		1.00 (reference)		
	GC	129 (18.7)	155 (15.2)	1.30 (1.00-1.68)	0.047	1.30 (1.00-1.68)	0.047	
	GG	10 (1.4)	6 (0.6)	1.61 (0.97-2.68)	0.066	1.62 (0.97-2.70)	0.065	
Dominant	CC	551 (79.9)	859 (84.2)	1.00 (reference)		1.00 (reference)		
	GC/GG	139 (21.4)	161 (15.8)	1.35 (1.05-1.73)	0.020	1.35 (1.05-1.73)	0.021	

OR: odd ratio; CI: confidence interval. CHD: Coronary heart disease. *Adjusted for age, gender, smoking status, and dyslipidemia.

Considering that hypertension, coronary heart disease, and diabetes are important risk factor for heart failure, association analyses stratified by these comorbidities were further performed. Univariate analysis showed that rs2306235 G allele was associated with increased risk of CHF in patients with hypertension and coronary heart disease (hypertension: OR =1.40, 95% CI =1.08-1.81, *p* = 0.011; coronary heart disease: OR = 1.37, 95% CI = 1.07-1.76, *p* = 0.014). Also, rs2306235 G allele was associated with increased risk of CHF in nondiabetic patients (OR = 1.35, 95% CI = 1.05-1.73, *p* = 0.020). After adjusting for age, gender, smoking status, and dyslipidemia, the association was still significant (hypertension: OR = 1.45, 95% CI = 1.09-1.75, *p* = 0.006; coronary heart disease: OR = 1.41, 95% CI = 1.09-1.83, *p* = 0.010; diabetes: OR = 1.59, 95% CI = 1.08-2.35, *p* = 0.019; nondiabetes: OR = 1.35, 95% CI = 1.05-1.73, *p* = 0.021). However, rs2306235 polymorphism was not associated with risk of CHF in patients without hypertension and coronary heart disease (Table 2).

### *CKIP-1* rs2306235 polymorphism and severity of CHF

Echocardiographic examination was performed only in 839 CHF patients. Comparisons of NYHA class, left ventricular ejection fraction (LVEF), and left ventricular end-diastolic diameter (LVEDD) among rs2306235 genotypes were presented in Table 3. No significant differences in NYHA class, LVEF, and LVEDD were observed among rs2306235 genotype groups (*p* = 0.743, 0.626, and 0.652, respectively).

**Table 3:** Association of *CKIP-1* rs2306235 polymorphism with severity of CHF

Variables	CC (n=736)	GC (176)	GG (n=11)	*P*
NYHA class				0.743
II, n (%)	256 (34.8)	61 (34.7)	3 (27.3)	
III, n (%)	273 (37.1)	70 (39.8)	6 (54.5)	
IV, n (%)	207 (28.1)	45 (25.6)	2 (18.2)	
LVEF (%)*	38.2 ± 9.2	37.5 ± 9.2	37.6 ± 10.4	0.626
LVEDD (mm)*	61.1 ± 7.6	61.7 ± 6.7	61.3 ± 8.6	0.652

NYHA: New York Heart Association, LVEF: left ventricular ejection fraction, LVEDD: left ventricular end-diastolic diameter. *Based on 839 patients with chronic heart failure.

### *CKIP-1* rs2306235 polymorphism and prognosis of CHF

**Figure 1:**
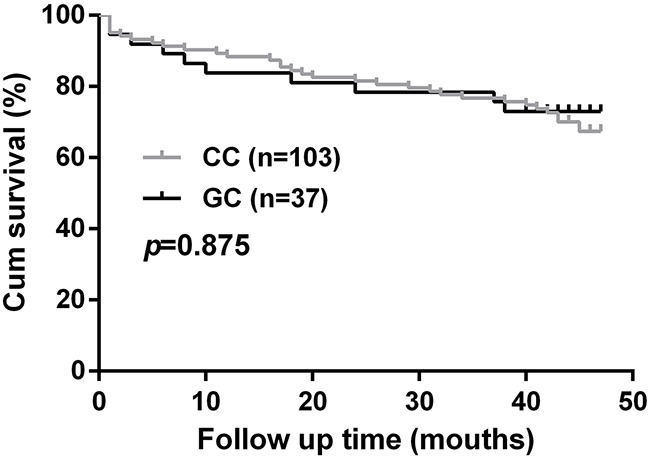
Association of *CKIP-1* rs2306235 polymorphism with cardiovascular mortality in chronic heart failure patients

A total of 140 patients were followed up for a median period of 38.7 months. No significant difference in cardiovascular mortality was observed between rs2306235 genotype groups (CC vs GC, *p* = 0.875, Figure 1).

## DISCUSSION

We firstly identified rs2306235 G allele associated with increased risk of CHF in the entire cohort. Stratified analyses demonstrated that the association was still significant in patients with hypertension and coronary heart disease regardless of diabetes mellitus. However, this polymorphism showed no association with severity and cardiovascular mortality in CHF patients.

Prolonged pressure stresses, including hypertension and myocardial infarction can lead to pathological cardiac hypertrophy and heart failure [24]. CKIP-1 can suppress pathological cardiac hypertrophy through promoting HDAC4 dephosphorylation by recruiting PP2A. [22]. *CKIP-1* deficient mice develop spontaneous cardiac hypertrophy with aging and hypersensitivity to pressure overload-induced cardiac hypertrophy [22]. In contrast, myocardial *CKIP-1* overexpression protects from pressure overload-induced cardiac hypertrophy [22]. Significant reduction of CKIP-1 protein level was also observed in the late phases of cardiac hypertrophy in mouse and human hearts [22]. Collectively, these data may suggest a inhibitory effect of CKIP-1 on heart failure caused by pathological cardiac hypertrophy.

Rs2306235 is a missense polymorphism (Pro 21 Ala) in *CKIP-1* exon 2, which encodes the N-terminal PH domain of CKIP-1 [7]. This PH domain is required for interaction and recruitment of multiple proteins to the plasma membrane, where CKIP-1 localizes predominantly and perform most of its known functions [23]. Functional prediction of this polymorphism was performed using PROVEAN, SIFT, and PolyPhen-2. PROVEAN and SIFT assessment of the proline to alanine amino acid substitution predicted “neutral” and “tolerated”, respectively; and PolyPhen-2 predicted this variant to be benign with a score of 0 (data not shown). However, its exact function deserves further investigation. Moreover, the MAF of rs2306235 polymorphism has ethnic difference. According to the 1000 Genomes Project, the MAF of rs2306235 ranges from 0.063 to 0.129 in East Asian population. However, the MAF is 0 in both American and African population. And also, rs2306235 is a rare polymorphism with MAF of 0.001 in the European population. Therefore, rs2306235 polymorphism may exhibit more significant association with CHF in East Asian population.

Considering that hypertension, coronary heart disease, and diabetes are important risk factors for heart failure [25], stratified analysis was further performed. Stratified analysis showed that rs2306235 polymorphism was significantly associated with risk of CHF in patients with hypertension and coronary heart disease. This association was also evident in both diabetic and nondiabetic heart failure, which indicated that rs2306235 polymorphism modify risk of CHF independent of diabetes. Left ventricular hypertrophy, the most visible manifestation of target organ damage in hypertension, eventually predisposes to heart failure [26]. Moreover, coronary heart disease also has a higher prevalence of left ventricular hypertrophy [27]. Given the crucial role of CKIP-1 in inhibiting cardiac hypertrophy, *CKIP-1* polymorphism may be a potential risk factor for CHF in patients with hypertension and coronary heart disease.

Several limitations of this study deserve consideration. First, we only enrolled Han Chinese participants with relatively limited sample size. As MAF of rs2306235 varies among different ethnicities, whether our results are generalized to other ethnicity still needs to be clarified. Second, only rs2306235 polymorphism was investigated in the present study, other potentially functional polymorphisms in *CKIP-1* should been evaluated for their association with CHF. In addition, fewer patients were followed up in a relatively short period of time. Further study with larger patients and long term follow up is needed. Finally, functional assessment of rs2306235 polymorphism was not performed. Therefore, further investigations are necessary to clarify the exact relationship between this variant and heart failure.

In conclusion, we firstly revealed the association of *CKIP-1* rs2306235 polymorphism with an increased risk of CHF in Chinese Han populations. Further investigations in larger cohorts may be required to verify our findings. More comprehensive survey and functional experiments are also needed to illuminate the exact mechanism behind our findings.

## MATERIALS AND METHODS

### Study participants

A total of 923 patients with CHF were recruited between November 2011 and December 2014 from Xiangya Hospital, Central South University. The eligibility criteria were described previously [5]. Patients with heart failure were eligible if they were aged 20-80 years and had chronic and stable heart failure symptoms, as demonstrated by New York Heart Association functional class II-IV, for more than three months prior to study initiation. The exclusion criteria were tumors or malignant disease, severe hepatic or renal dysfunctions, and pregnancy. The etiology of heart failure included ischemic heart disease and nonischemic cardiomyopathy. Ischemic heart disease was defined by satisfying one of the following conditions: ≥ 50% luminal stenosis of at least one major coronary artery, history of myocardial infarction ≥ three months before enrolment, history of coronary artery bypass graft, and coronary stent implantation. Patients were contacted by a questionnaire during regular outpatient clinics or by a structured telephone interview. Data concerning cardiovascular mortality during the follow-up period were collected.

A group of 1020 age- and gender-matched healthy controls were also enrolled in physical examination center of the same hospital. The subjects were apparently healthy as assessed by physical examination, serum biochemical testing, and electrocardiogram. Hypertension was defined as participants with a systolic blood pressure (SBP) ≥ 140 mmHg and/or a diastolic blood pressure (DBP) ≥ 90 mmHg or those who received at least one antihypertensive medication. Coronary heart disease (CHD) was defined as luminal stenosis ≥ 50% in at least one major coronary artery branch or myocardial infarction. Diabetic mellitus was diagnosed as a fasting plasma glucose ≥ 7.0 mmol/L or under diabetes medication. The healthy controls had no histories or symptoms of hypertension, CHD, diabetes mellitus, or any other cardiovascular diseases.

All subjects were Han Chinese origin as ascertained by their resident identity card. The study protocol was approved by the ethic committee of School of Pharmaceutical Sciences, Central South University and registered in Chinese Clinical Trial Registry (registration number: ChiCTR-RCC-12002817). Informed consent was obtained from each participants prior to enrollment.

### Polymorphism selection and genotyping

Based on the CHB population (Han Chinese in Beijing, China) of 1000 Genomes project (http://browser.1000genomes.org), a total of seven tag polymorphisms with minor allele frequency (MAF) > 0.05 were identified using Haploview 4.2 software. Among them, only rs2306235 polymorphism is located in *CKIP-1* exon. This polymorphism results in replacement of proline by alanine at the 21 amino acid (Pro21Ala). This amino acid is located in the CKIP-1 PH domain [7], which is required for mediating multiple interactions between CKIP-1 and numerous cellular proteins [8]. Therefore, rs2306235 polymorphism was selected in our study.

Venous blood samples were collected into EDTA tube and stored at -20°C. Genomic DNA of each subject was extracted from peripheral blood leukocytes using the standard phenol/chloroform protocol. *CKIP-1* rs2306235 polymorphism was genotyped by PCR-restriction fragment length polymorphism. The target fragment of 425 bp was amplified using primers 5′-tgaaaacctttccgaagtgg-3′ and 5′-accactgaacttgcctttgg-3′ (forward/reverse). Five microliters of PCR products was digested with *Pst* I restriction enzyme (Thermo Fisher Scientific, Waltham, MA, USA) overnight at 37°C. The digested PCR products were then analyzed on an agarose gel followed by ethidium bromide staining. Additionally, a random selection of 5% of the samples was also genotyped by Sanger sequencing with the ABI PRISM 3730XL DNA sequencer (Applied Biosystems, Foster City, CA, USA) and the genotyping were confirmed in 100%.

### Statistical analysis

Continuous values were expressed as mean ± SD and categorical variables as numbers and percentages. Patient characteristics were compared using Chi-square test for categorical variables and independent sample t test or one way analysis of variance for continuous variables. Hardy-Weinberg equilibrium of genotypic distribution was evaluated using a Chi-square test. The association between *CKIP-1* polymorphism and the risk of CHF was estimated by unconditional logistic regression analysis adjusting for multiple cardiovascular risk factors. The effect of *CKIP-1* polymorphism on disease survival was estimated using Kaplan-Meier curve and Cox proportional hazards regression model. The statistical analysis was performed with SPSS software (version 13.0, SPSS Inc., Chicago, IL, USA). A two-sided *p* value < 0.05 was considered to be statistically significant.

## Abbreviations

CHF: chronic heart failure
CKIP-1: casein kinase-2 interacting protein-1
PH domain: pleckstrin homology domain
HDAC4: dephosphorylation of histone deacetylase 4
SBP: systolic blood pressure
DBP: diastolic blood pressure
TC: total cholesterol
LDL-C: low-density lipoprotein cholesterol
HDL-C: high-density lipoprotein cholesterol
MAF: minor allele frequency
CHD: coronary heart disease
LVEF: left ventricular ejection fraction
LVEDD: left ventricular end-diastolic diameter
